# Temperature-Sensitive Template for Preparation of ZnO/CeO_2_ Composite Photocatalytic Materials and Its Catalytic Performance

**DOI:** 10.3390/molecules29153589

**Published:** 2024-07-30

**Authors:** Yaoyao Zhang, Wenjie Yang, Zhengyuan Zhu, Lin Zhang, Wenju Peng

**Affiliations:** 1 School of Chemistry and Materials Science, Hubei Engineering University, Xiaogan 432000, China; 15394082645@163.com (W.Y.); zhengyuanzhu@foxmail.com (Z.Z.); 13415968580@163.com (L.Z.); 2 School of Materials Science and Engineering, Hubei University, Wuhan 430062, China; 3 School of Civil Engineering, Hubei Engineering University, Xiaogan 432000, China

**Keywords:** Ce-doped ZnO, temperature sensitive template, photodegradation, methyl orange, reuse

## Abstract

In this work, a series of thermosensitive ionic liquid functionalized polymers, PN_x_(IL)_y_, with controllable morphology and particle size were prepared by free radical polymerization. Then, using the polymer PN_64_(IL)_8_ with uniform morphology as a templating agent, the ZnO composite photocatalytic materials doped with rare earth metal Ce were prepared in combination with a microwave-assisted and templated hydrothermal reaction method. Series different Ce-doping amount photocatalytic materials ZnO-Ce-x‰ were characterized by XRD, SEM, TEM, XPS, and other methods. The results demonstrated that the templated materials PN_64_(IL)_8_ can prepare ZnO-Ce-2‰ with uniform petaloid ambulacra shape, good distribution of elements, and excellent photocatalytic performance. Photocatalytic degradation experiments of methyl orange (MO) showed that when the Ce-doping amount is only 2‰, the degradation rate of organic dyes can reach 96.5% by reacting the photocatalytic materials in water for 1 h. In addition, this kind of photocatalyst can be used for the degradation of high-concentration MO, as well as being easily recovered and effectively reused by simple filtration. Therefore, the structure of this kind of photocatalyst is controllable in the preparation process with an extremely low Ce-doping amount compared with current reports, and it has a good application prospect in the field of wastewater treatment technology.

## 1. Introduction

Organic dyes are one of the important components in textiles, optoelectronics, food, cosmetics, and other fields [1]. They are usually insoluble in water, and easily cause industrial organic wastewater, resulting in environmental pollution [2,3]. To solve this problem, researchers have been working hard to find effective ways to remove organic pollutants. Up to now, the organic dyes in industrial wastewater are mainly treated by chemical coagulation, precipitation, adsorption, and membrane filtration technologies [4,5]. However, these methods have the disadvantages of low efficiency and high cost.

As a semiconductor-based photocatalyst, ZnO nanoparticles have been widely reported for the removal of organic dyes, which have the advantages of low cost, good biocompatibility, simple preparation method, and can be reused many times [6,7,8]. Although it has been reported that ZnO can degrade Rhodamine B with a degradation rate of 90%, it can also be used as an effective photocatalytic material to decompose methylene orange, Nile blue, and other organic dyes [9,10,11]. However, the photocatalytic efficiency of pure ZnO is strongly influenced by the particle size, morphology, and specific surface area. In addition, ZnO nanoparticles are prone to agglomeration in an aqueous solution, resulting in a rapid decrease in surface area and hence catalytic efficiency. Therefore, it is urgent to modify ZnO nanoparticles to enhance their photocatalytic activity. However, ZnO has the problems of low light absorption efficiency, high e--h+ recombination rate, and photo corrosion. To solve these problems, researchers have done a lot of work to explore the modification of ZnO, including control morphology, semiconductor recombination, ion doping, precious metal deposition, and so on. In many studies, doped ZnO has shown better photocatalytic performance, and the dopant can be metal, such as Mn [12,13,14], Fe [15,16], Ni [17,18], Ag [19,20,21], Ce [22,23], or nonmetal C [24,25], and N [26,27,28]. Among all kinds of doping, Ce doping can significantly enhance ZnO carriers and thus improve photocatalytic activity. Despite the optical and electrical properties of Ce-doped ZnO in the literature, studies on the photocatalysis performance of ZnO/CeO_2_ nanocomposites remain relatively scarce. It has been revealed that ZnO/CeO_2_ nanocomposites possess exceptional properties suitable for diverse applications, including catalysis [29], biomedical [30], and antibacterial applications [31].

In preparing Ce-doped ZnO, the difficulty lies in how to obtain the material with good morphology and uniform loading. Based on our existing research foundation, considering the use of the template hydrothermal reaction method [32], we can prepare photocatalytic materials with excellent morphology and performance by using a suitable template. In our previous study, the temperature-sensitive ionic liquid functional polymer PN_x_(IL)_y_ is a kind of water-soluble polymer that can self-assemble into nanoparticles with regular morphology and structure in water, and it has been reported to be applied in the field of catalysis [33]. Therefore, we can choose this kind of polymer, PN_x_(IL)_y_, combined with the template hydrothermal method to prepare excellent photocatalytic materials.

In this study, a series of temperature-sensitive ionic liquid functional polymers (PN_x_(IL)_y_) were prepared by RAFT polymerization. Using the polymer with regular morphology and uniform particle size as the template agent, the rare earth metal Ce-doped ZnO composite photocatalytic materials were prepared by microwave-assisted and template hydrothermal reaction methods. The series characterization shows that Ce is successfully doped into the ZnO structure and the morphology is good. Finally, it was applied to the degradation of organic dyes, and the catalytic performance, kinetic reaction performance, and recovery performance of the materials were investigated.

## 2. Results and Discussion

### 2.1. XRD of the Ce-Doped ZnO Photocatalysts

**Figure 1:**
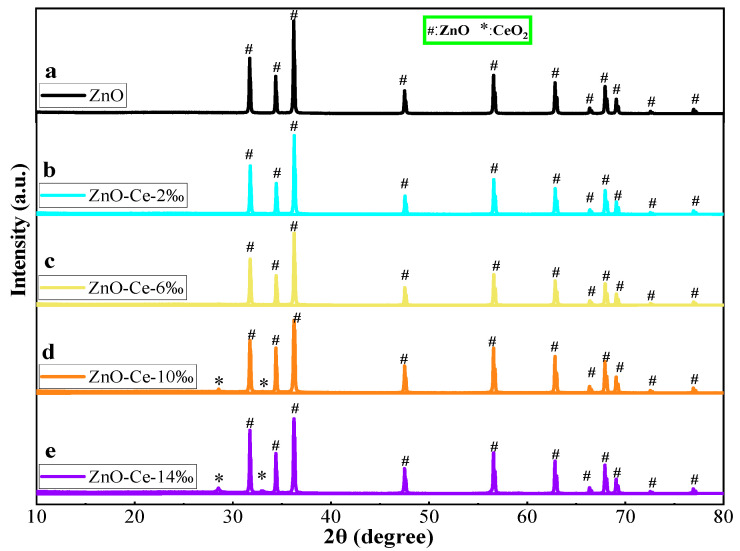
XRD spectra of ZnO (**a**), ZnO-Ce-2‰ (**b**), ZnO-Ce-6‰ (**c**), ZnO-Ce-10‰ (**d**), ZnO-Ce-14‰ (**e**).

Figure 1 shows the XRD pattern of ZnO and different Ce amount of photocatalytic materials ZnO-Ce-x‰. It can be seen from the results that ZnO peaks appear at 2θ 31.72°, 34.34°, 36.21°, 47.65°, 56.55°, 62.79°, 66.51°, 67.81°, and 69.02°, respectively. The diffraction peaks correspond to (100), (002), (101), (102), (110), (103), (200), (112), and (201) crystal faces of hexagonal wurtzite [34]. The radius of Ce^4+^ (0.103 nm) was larger than that of Zn^2+^ (0.074 nm), so it was difficult for Ce^4+^ to enter the ZnO lattice by substituting Zn^2+^ (CeZn) or filling zinc vacancy (V_Zn_) [35]. Relevant studies have shown that Ce^4+^ could be bonded to the surface of ZnO crystals by chemical bonding of Zn-O-Ce [36]. When using PN_64_(IL)_8_ as a template, the XRD pattern of the Ce-doped ZnO composite photocatalytic material showed the same characteristic peaks as that of the ZnO standard diagram. The same peaks indicate that Ce doping did not affect the typical structure of ZnO, and the crystal surface remains intact. With the increase in metal Ce content, the characteristic diffraction peak gradually showed at 28.56° and 32.97°. The new characteristic peak corresponds to the (111) crystal face of CeO_2_ of the cubic cerium mineral phase. In addition, the composite photocatalytic material did not change the characteristic structure of the original ZnO crystal after Ce doping. The (111) crystal face of CeO_2_ can observe the crystal face characteristics of Ce more in ZnO-Ce-10‰ and ZnO-Ce-14‰, which was marked by “*” at 28.56° and 32.97°.

### 2.2. SEM of the Ce-Doped ZnO Photocatalysts

**Figure 2:**
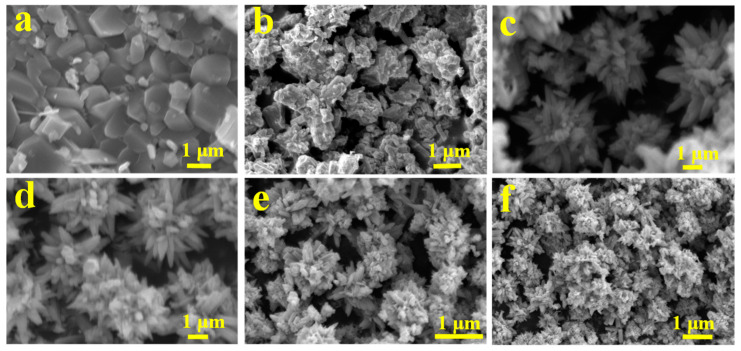
SEM of ZnO (**a**) without template PN_64_(IL)_8_, and ZnO (**b**), ZnO-Ce-2‰ (**c**), ZnO-Ce-6‰ (**d**), ZnO-Ce-10‰ (**e**), ZnO-Ce-14‰ (**f**) with the added template PN_64_(IL)_8_.

We investigated the effect of the templating agent PN_64_(IL)_8_ on the morphology of Ce-doped ZnO composite photocatalytic materials by SEM characterization. The results are shown in Figure 2. Without the templating agent PN_64_(IL)_8_, the pure ZnO prepared by the microwave-assisted combined hydrothermal method had no regular morphology, and showed sheet structures with different sizes and small specific surface area. After the addition of PN_64_(IL)_8_, ZnO grows into three-dimensional morphology material and the specific surface area increases. ZnO-Ce-2‰ photocatalytic materials have been prepared by adding Ce under the action of template agent PN_64_(IL)_8_. The prepared ZnO-Ce-2‰ showed a regular and uniform petal-like morphology, and the specific surface area of ZnO-Ce-2‰ was larger with a diameter of about 4 μm. When the doping amount was 6‰, ZnO-Ce-6‰ still showed a regular petal-like morphology. While further increasing the Ce amount, the obtained ZnO-Ce-10‰ and ZnO-Ce-14‰ materials showed petal-like imperfections. The imperfections were probably caused by the chemical bonding of metal Ce to form the Zn-O-Ce and ZnO crystal surfaces, and the petal-like surface grows CeO. Therefore, under the role of template agent PN_64_(IL)_8_ with the Ce-doping amount between 2‰ and 6‰, the petal-like materials with regular morphology and large specific surface area can be obtained.

### 2.3. TEM and TEM Mapping of the ZnO-Ce-2‰ Photocatalyst

**Figure 3:**
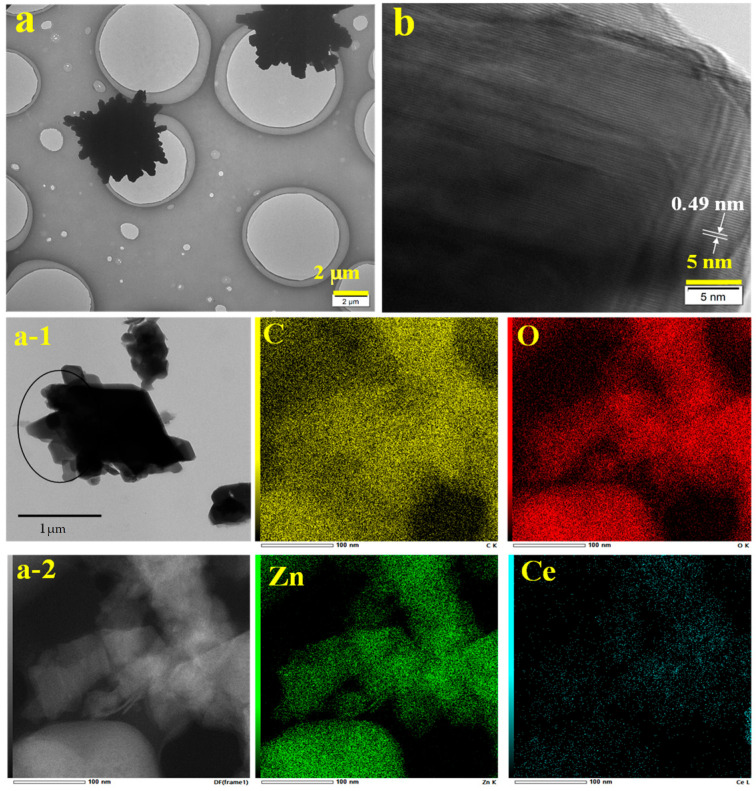
TEM (**a**), HRTEM (**b**), and TEM mapping (a-1, a-2, C, O, Zn, Ce) of ZnO-Ce-2‰ with the added template PN_64_(IL)_8_.

As shown in Figure 3, the morphological features of ZnO-Ce-2‰ were further analyzed by TEM and HRTEM. As the same with SEM, the TEM image also exhibits ZnO-Ce-2‰ with regular and uniform petal-like morphology, and the diameter was about 4 μm. In the HRTEM characterization, only one fringe structure with a lattice spacing of 0.49 nm was observed, which belongs to the lattice fringe of the wurtzite ZnO structure [37]. The lattice structure of Ce in the ZnO-Ce-2‰ structure was not observed, which may be due to the low doping amount and that Ce does not form a good crystal structure on the surface of the ZnO structure.

To further prove Ce was successfully doped into the ZnO structure, the ZnO-Ce-2‰ was characterized by TEM mapping. As Figure 3 shows, Zn and Ce were evenly distributed in the ZnO-Ce-2‰ photocatalyst by scanning the element content in the petal structure.

### 2.4. XPS of the ZnO-Ce-2‰ Photocatalyst

**Figure 4:**
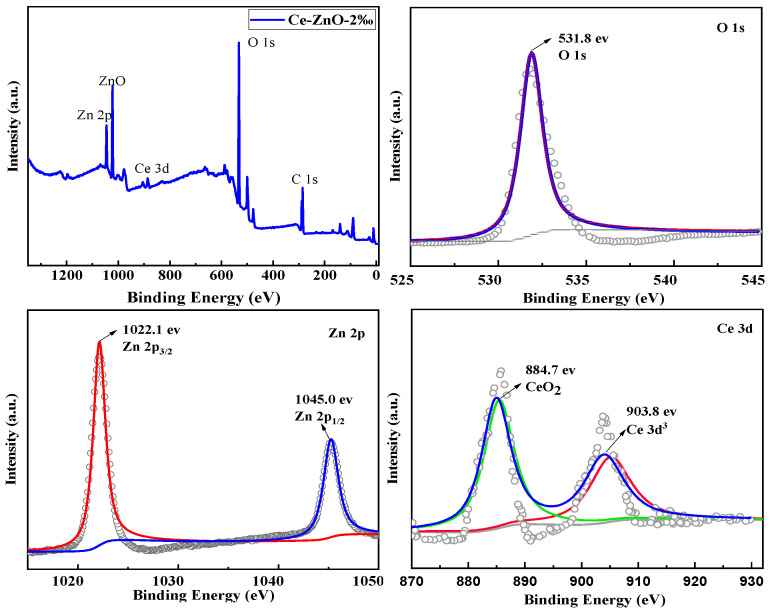
XPS of ZnO-Ce-2‰ with the added template PN_64_(IL)_8_.

In order to investigate the chemical composition and valence states of the elements, the ZnO-Ce-2‰ photocatalytic material was tested by XPS. As shown in Figure 4, O, Zn, and Ce in ZnO-Ce-2‰ materials belong to ZnO and CeO_2_, respectively. The binding energy of the C 1s peak was used to calibrate the baseline. Figure 4 shows that the binding energy of Zn in ZnO-Ce-2‰ material is around 1022.1 eV and 1045.0 eV, and the high-resolution XPS spectra of Zn 2p were perfectly fitted, so Zn in the sample exists in the form of Zn^2+^ [38]. The Ce 3d photoelectron peak could be divided into two pairs of spin-orbit multiplets, with the peak values at 884.7 eV and 903.8 eV, confirming the existence of Ce^4+^ [39]. To better understand the content of Ce element, we performed XPS analysis on all materials of ZnO-Ce-6‰, ZnO-Ce-10‰, and ZnO-Ce-14‰ and provided the atomic content percentage in Supplementary Materials. As Raman spectra in Supplementary Materials, the *E*_1_(LO) band at 582.8 of ZnO-Ce-2‰ is a manifestation of the resonant enhancement of the LO mode due to the presence of Ce-doping defects. The systematic increase in the $E_{1}(LO)$/$E_{2}^{\mathrm{high}}$ is in accord with an increasing defect concentration with the adding of Ce to form ZnO-Ce-2‰ ($E_{1}(LO)$/$E_{2}^{\mathrm{high}}$ = 0.2115) than ZnO ($E_{1}(LO)$/$E_{2}^{\mathrm{high}}$ = 0.0888) by calculation [40,41].

### 2.5. Catalytic Performance of Photocatalysts

**Figure 5:**
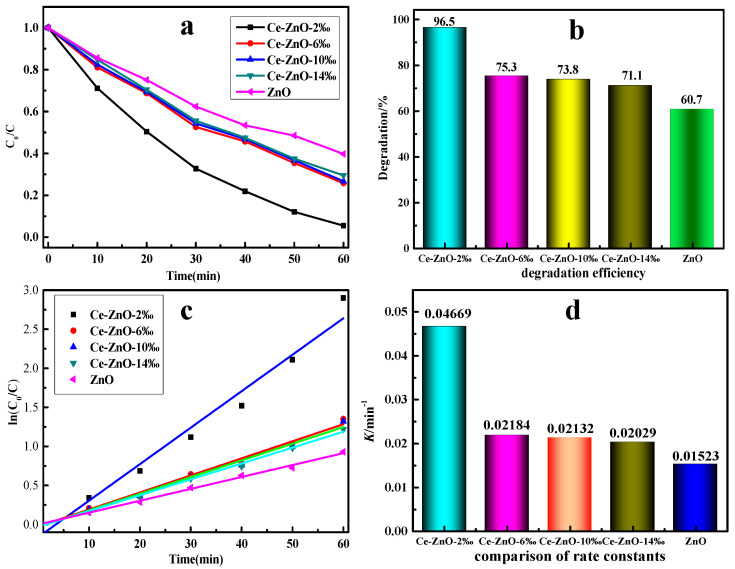
Photocatalytic degradation (**a**), degradation efficiency (**b**), kinetics of photocatalytic degradation (**c**), and comparison of rate constants for the different prepared samples (**d**).

Figure 5 shows the effect of photocatalytic material prepared in the presence of template agent PN_64_(IL)_8_ on the degradation of MO solution. It can be seen that the photodegradation efficiency of ZnO prepared by the template agent without Ce was only 60.7%, while the photodegradation efficiency of MO can be significantly improved after Ce doping. When PN_64_(IL)_8_ was used as a template agent, the efficiency of the ZnO-Ce-2‰ composite photocatalytic material can be greatly improved even if the doping concentration was only 2‰. The conversion rate could reach to 96.5% after 60 min of reaction. The catalytic efficiency of ZnO-Ce-6‰ decreased to 75.3%, and ZnO-Ce-10‰ maintained at 73.8% (Figure 5a,b). As the Ce content continued to increase, the degradation efficiency of the composite photocatalyst decreased. The decreased efficiency was mainly due to the excessive addition of Ce ions which destroy the hexagonal wurtzite ZnO structure, thus affecting the photodegradation efficiency. Therefore, under the condition of PN_64_(IL)_8_ as a template agent, the microwave-assisted hydrothermal method could be used to obtain the composite photocatalytic material. Additionally, excellent morphology and excellent photocatalytic efficiency were obtained when the doping amount was only 2‰ at low temperature (120 °C).

To further understand the kinetic characteristics of the reaction process, the degradation process was fitted linearly. It was found that the degradation was in accordance with the first reaction rate equation, and the linear correlation was good (Figure 5c). Among the reaction rate constants, the rate constant of the photocatalytic material ZnO-Ce-2‰ was the highest with 0.04669, about twice that of other photocatalytic materials. The highest catalytic efficiency of ZnO-Ce-2‰ was probably due to the best petal-like shape with a large specific surface area. The rate constant of pure ZnO photocatalytic materials was only 0.01523, which was lower than that of other Ce-doped photocatalytic materials (Figure 5d). The above kinetic results show that doping Ce for ZnO could significantly improve the degradation rate of MO [42,43]. 

### 2.6. Time-Dependent ZnO-Ce-2‰ for MO

**Figure 6:**
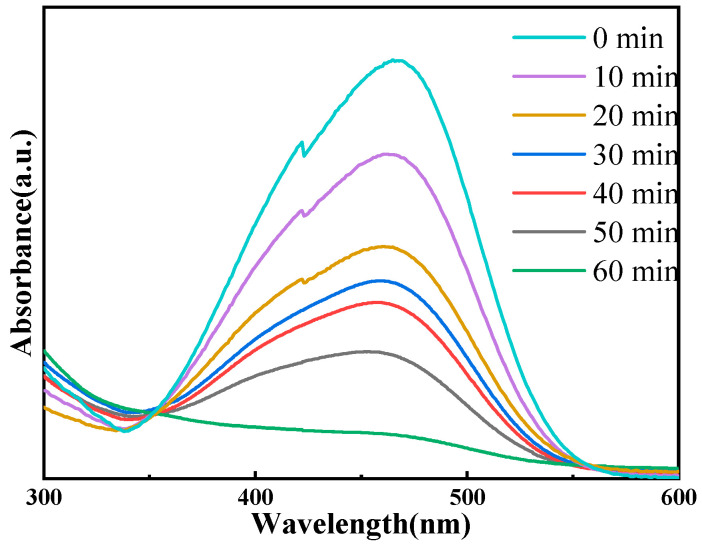
The time-dependent UV-visible absorption spectra of MO in the presence of ZnO-Ce-2‰.

The photocatalytic performance test model was conducted with ZnO-Ce-2‰ as the catalytic material and MO as the degradation material. Kinetic tests were conducted with samples taken every 10 min, and the results are shown in Figure 6. It can be seen from the kinetic experiment that the MO intensity at 464 nm decreases with time, and the degradation rate reaches the highest of 96.5% at 60 min. Moreover, the initial concentrations of MO were then investigated, and MO solutions with concentrations of 10 mg/L, 15 mg/L, 20 mg/L, 25 mg/L, and 30 mg/L were prepared. The results were shown in Supplementary Materials. Even at a higher concentration of 25 mg/L, the degradation rate is up to 75.3%. This work was higher than the general MO concentration reported in the current literature, and the catalytic degradation effect was much better [44,45,46,47].

### 2.7. Reuse of ZnO-Ce-2‰ for MO

**Figure 7:**
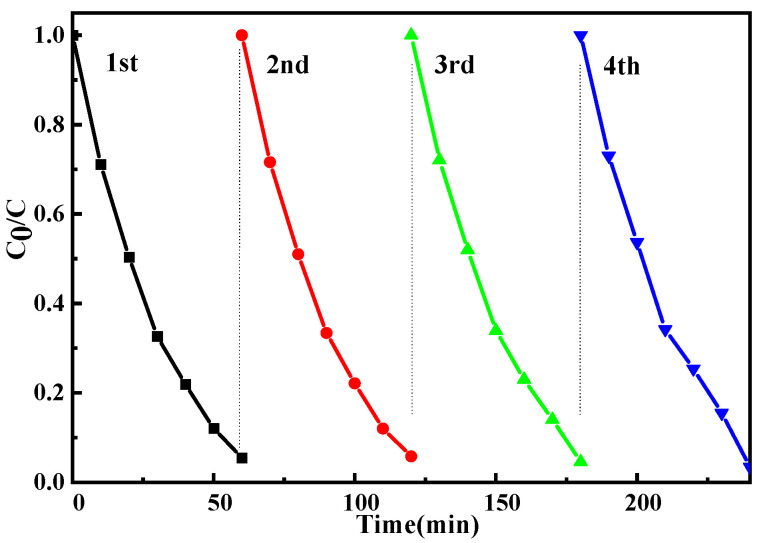
The reuse of ZnO-Ce-2‰ for MO degradation.

To test the industrial performance of the catalytic material, the recycling test was carried out. After the reaction, a centrifuge at 8000 rpm was used for 5 min, and the precipitate in the lower layer was washed and dried with deionized water for the next reuse. As shown in Figure 7, after 4 cycles, the degradation rate of MO was still at a high level of more than 90%. Therefore, according to the results of repeated use experiments, the photocatalytic material ZnO-Ce-2‰ prepared by using water-soluble temperature-sensitive material as a template and microwave-assisted hydrothermal method has good catalytic degradation performance of MO. What is more, ZnO-Ce-2‰ still exhibits good catalytic degradation performance when soaked in MO solution for a long time. The Ce-doped ZnO photocatalytic material prepared by this new method has a series of advantages in the field of wastewater treatment, such as low doping amount, less dosage, stable material properties, and has a good prospect for industrial application. To prove the stability of ZnO-Ce-2‰, we carried out the SEM characterization of the reuse for ZnO without Ce doping. The results showed that the SEM morphology of pure ZnO was changed after the UV photocatalytic reaction, indicating that ZnO suffers from photocorrosion. After 4 cycles of photocatalytic reaction, the morphology of ZnO-Ce-2‰ was characterized, and it was found that the SEM morphology had no change. After recovery, XRD and XPS remained unchanged, and the characteristic peaks were still present (see Supplementary Materials). The results show that the addition of Ce element can protect the morphology of zinc oxide and attenuate its photocorrosion [48].

### 2.8. Mechanism of ZnO-Ce-2‰ for MO Degradation

**Figure 8:**
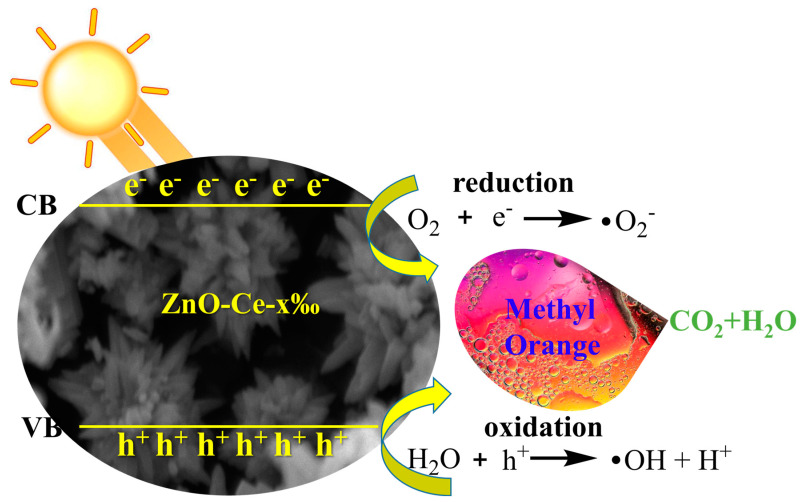
Schematic illustration of the photocatalytic mechanism of ZnO-Ce-2‰ for MO degradation.

The valence band of ZnO produced an excited e- due to the absorption of UV energy, which transitions to the ZnO conduction band and leaves a h^+^ in the valence band. After e^-^ and h^+^ were excited to transfer to the ZnO crystal surface or the ZnO-Ce-x‰ crystal surface through ZnO, they can react with O_2_, H_2_O, and OH^-^ in solution, respectively. Then, they generate photocatalytic •O_2_^−^ free radicals and •OH free radicals [49,50]. The doped Ce^3+^ was finally attached to the surface of ZnO crystals in the form of Ce^4+^, and CeO_2_ had a strong oxygen absorption capacity, which increases the O_2_ content near the ZnO crystals. On the one hand, the consumption of photogenerated e^-^ could effectively reduce the photogenerated e^−^-h^+^ pair recombination in ZnO crystals. On the other hand, with the increase in •O_2_^−^ and •OH active groups, the photocatalytic activity of the ZnO catalyst was improved (Figure 8). In addition, under high-concentration doping, the ZnO crystal structure will be slightly distorted, resulting in more V_O_, V_Zn_, and other defects, which may become new e^−^-h^+^ composite centers, resulting in reduced photocatalytic activity of ZnO. Therefore, ZnO-Ce-2‰ performed the highest catalytic degradation efficiency.

## 3. Materials and Methods

### 3.1. Materials

Zinc salt (ZnAc_2_·2H_2_O), sodium hydroxide, cerium chloride heptahydrate (CeCl_3_·7H_2_O), citric acid, N-isopropyl acrylamide (NIPAAm), vinyl imidazole, and methyl orange (MO) were purchased from Picard medical. Azobisisobutyronitrile was purchased from Shanghai Civic Chemical (Shanghai, China). Other commercially available chemicals were laboratory-grade reagents from local suppliers. All solvents were purified by standard procedure. Column chromatography silica gel and thin-layer silica gel were produced by Qingdao Ocean Chemical Silica Gel Factory (Qingdao, China). The synthesis of vinyl imidazole ionic liquid is referred to in the literature [33].

### 3.2. Analytical Methods

In gel permeation chromatography (GPC, Alltech, Lexington, KY, USA) analyses, THF was used as a solvent eluting at a flow of 1 mL/min with polystyrene standards as calibration, and the detection temperature is 40 °C with a column temperature of 30 °C through a Jordi GPC 10000 A column (300 mm × 7.8 mm) (Mansfield, MA, USA) equipped with an Alltech ELSD 800. FT-IR spectras were obtained using an AVATAR 370 Thermo Nicolet spectrophotometer (Nicolet, Green Bay, WI, USA). XRD patterns were recorded on a Bruker-D8 diffractometer with Cu Kα radiation (λ = 0.15406 nm) to evaluate the crystal structure of ZnO-Ce-x‰ samples (Bruker, Rheinstetten, Germany). The particle size and the morphology of the ZnO-Ce-x‰ were observed by SEM (Hitachi S3400N, Hitachi, Tokyo, Japan) and TEM (FEI Tecnai G20, FEI, Hillsboro, OR, USA). The XPS analysis was carried out on a Thermo Fisher Scientific K-Alpha spectrometer (Waltham, MA, USA) using monochromatic Al-Ka radiation at a detection angle of 30° (Invitrigen, Waltham, MA, USA). The photochemical reaction apparatus (GHX-IV, Shanghai, China) was used as a photocatalytic material for the degradation of MO. UV-Vis Agilent 8453 was used to test the absorbance of MO solution (Agilent, Santa Clara, CA, USA).

### 3.3. Preparation of Temperature-Sensitive Template PN_64_(IL)_8_

Vinylimidazole ionic liquid compounds were prepared by the 1:1 reaction of vinylimidazole with bromoethane as described in the literature [51,52]; 10 mmol of vinyl imidazole and 10 mmol of chloromethane were added to 20 mL ethanol, forming a colorless solution that was refluxed under nitrogen for 24 h. After the full reaction, the resulting mixture was purified by re-crystallization from ethanol, with ethyl acetate as an anti-solvent, and named IL. Then, 6.4 mmol of NIPAAm, 0.8 mmol of IL, 1/6 mmol of thiopropionic acid benzoic acid ester, and 1/30 mmol of azodiisobutyronitrile (AIBN) were dissolved in methanol and the solution was added to Schlenk tube. The reaction was carried out at 60 ℃ for 24 h under N_2_ protection. Then, concentrated reaction liquid was under a vacuum and the polymer was repeatedly precipitated with several times of excess ether to obtain a light yellow product. After vacuum drying at 30 °C, the fluffy light-yellow polymerization product PN_64_(IL)_8_ was obtained. PN_64_(IL)_8_: FT-IR (KBr): γ_max_/cm^−1^ 3293, 3067, 2972, 2937, 2874, 1648, 1543, 1463, 1388, 1365, 1271, 1253, 1206, 1173, 1131, 1028, 924, 868, 850, 756, 709, 647, 587, 489 cm^−1^. GPC (THF): *M_n_* = 9581, *M_w_* = 10372, PDI = 1.08.

### 3.4. Preparation of Nanometer ZnO Materials Doped with Ce in Different Proportions

The different contents of Ce-doped ZnO photocatalysts were prepared by hydrothermal reactor method using PN_64_(IL)_8_ as a template (Table 1); 0.01 g PN_64_(IL)_8_ was dissolved in water, then ZnAc_2_·2H_2_O (0.998 mmol), citric acid (0.735 mmol), and CeCl_3_·7H_2_O (0.002 mmol) were added to the above solution under stirring circumstances until all reagents were completely dissolved. Microwaved for 0.5 h, and transferred to hydrothermal reactor at 120 °C for another 10 h. Cooled to room temperature, the obtained precipitate was centrifuged at 8000 r/min and washed at least three times with water to remove residual salts. Dried overnight in a vacuum drying oven at 50 °C, giving a white powder of ZnO-Ce-2‰. The references [53,54] show that interstitial incorporation is favored at low Ce concentrations, while substitution and interstitial substitution are comparable processes at high Ce concentrations. Based on economic principles, four different ratios of Ce-doped ZnO photocatalysts were prepared, and the doping concentrations of Ce were in mole percent: 0, 0.002, 0.006, 0.010, and 0.014 Ce-doped ZnO, respectively. The series photocatalysts were named ZnO-Ce-2‰, ZnO-Ce-6‰, ZnO-Ce-10‰, and ZnO-Ce-14‰, respectively (Table 1).

### 3.5. Photocatalytic Tests with Different Photocatalysts and Influence of Initial Concentration of MO

Next, 20 mg/L MO solution was accurately prepared, and 10 mg powder of different photocatalytic materials of ZnO-Ce-x‰ were weighed, respectively. ZnO-Ce-x‰ was added to 100 mL 20 mg/L MO solution, stirred, and dispersed for 30 min in the dark so that MO was completely pre-adsorbed on the catalyst surface; 10 mL of the solution was centrifuged at high speed and the supernatant solution was retained. The remaining solution was placed in the photocatalytic reaction apparatus, and the photodegradation experiment was carried out under the UV lamp at 500 W light with circulating water. The sample was taken at regular intervals and the supernatant was separated at high speed. UV spectrophotometer with the best detection wavelength (about 464 nm) was used to scan the 20 mg/L MO solution and measure the absorbance of the above two supernatants at the best detection wavelength. The degradation rate of the MO solution of the catalytic material was calculated by using the degradation rate formula. Then MO solutions of 10 mg/L, 15 mg/L, 20 mg/L, 25 mg/L, and 30 mg/L were prepared, and 10 mg of the catalytic material ZnO-Ce-2‰ powder was added to 100 mL of MO solution of different concentrations. All of the other steps were the same as above. The degradation rate of MO solutions was calculated to assess the influence of the initial concentration of MO. The following Formula (1) was used to calculate the degradation rate.

D = (A_0_ − A_t_)/A_0_ × 100% (1)

In the formula, D is the degradation rate; A_0_ is the initial absorbance value of MO solution; A_t_ is the photocatalytic time absorbance value of MO solution [55].

The formula used to calculate the reactivity values was the first-order kinetic reaction, which is represented by the mathematical expression as mentioned in Equation (2). By rearranging the above formula, we calculated the value of K (min^−1^), which shows the reactivity of a photocatalyst, or commonly known as the photodegradation constant [56].

(2) $$\ln(\frac{C_{0}}{C})=Kt$$

C_0_ is the initial concentration of MO solution (mg L^−1^), and C_t_ is the instant concentration of MO solution (mg L^−1^) at the time of the moment.

## 4. Conclusions

A novel series of polymers PN_x_(IL)_y_ were prepared, and the polymer with uniform morphology PN_64_(IL)_8_ was chosen as the template agent. Series different Ce-doping amount photocatalytic materials ZnO-Ce-x‰ have been successfully designed and prepared. Then, ZnO-Ce-x‰ were sufficiently characterized by XRD, SEM, TEM, and XPS to determine the morphology. Characterization results proved ZnO-Ce-2‰ with a uniform petaloid ambulacra shape and good distribution of elements. This kind of ZnO-Ce-x‰ catalyst could exhibit good performance compared with ZnO without a template agent. Remarkably, the photocatalyst with a lower Ce amount for ZnO-Ce-2‰ acquired the highest catalyst efficiency. The degradation rate of organic dyes can reach 96.5% by reacting with the photocatalytic materials in water for 1 h. Moreover, the ZnO-Ce-x‰ catalysts could be facilely recovered from the MO solution system for four cycles by simple centrifuge. The controllable preparation, high activity, as well as outstanding reusability, pave a green and efficient way for wastewater treatment in a green system.

## Figures and Tables

**Table 1:** Material ratio of synthesized ZnO-Ce-x‰ composite photocatalytic materials.

Entries	PN_64_(IL)_8_ (g)	n(Ce^3+^) (mmol)	n(Zn^2+^) (mmol)	Citric Acid (mmol)
ZnO	0.01	0	1	0.750
ZnO-Ce-2‰	0.01	0.002	0.998	0.735
ZnO-Ce-6‰	0.01	0.006	0.996	0.720
ZnO-Ce-10‰	0.01	0.010	0.990	0.705
ZnO-Ce-14‰	0.01	0.014	0.986	0.690

## Data Availability

Data are contained within the article.

## Supplementary Materials

The following supporting information can be downloaded at: https://www.mdpi.com/article/10.3390/molecules29153589/s1, Figure S1: Characterization of the template PN_64_(IL)_8_; Figure S2: XPS of ZnO-Ce-6‰, ZnO-Ce-10‰, ZnO-Ce-14‰ with the added of template PN_64_(IL)_8_; Figure S3: Raman spectra of ZnO (a), ZnO-Ce-2‰ (b), ZnO-Ce-6‰ (c), ZnO-Ce-10‰ (d), ZnO-Ce-14‰ (e); Figure S4: Influence of catalyst amount (a) and initial concentration of MO (b) in the presence of ZnO-Ce-2‰; Table S1: Atomic percentages of ZnO-Ce-x‰; Table S2: Raman modes of ZnO nanospindles in samples ZnO, ZnO-Ce-2‰, ZnO-Ce-6‰, ZnO-Ce-10‰, ZnO-Ce-14‰ [57].
