# Supplementary material for: Temperature-Sensitive Template for Preparation of ZnO/CeO2 Composite Photocatalytic Materials and Its Catalytic Performance
Source: Molecules. 2024 Jul 30;29(15):3589. doi: 10.3390/molecules29153589 (PMC11313708; doi:10.3390/molecules29153589)
Supplement: Supplementary file 1 [file molecules-29-03589-s001.zip › molecules-3104113-supplementary.pdf]

Supporting Information

Temperature-Sensitive Template for Preparation of ZnO/CeO_2 Composite Photocatalytic Materials and Its Catalytic Performance

Yaoyao Zhang [1,2,*], Wenjie Yang [1], Zhengyuan Zhu [1], Lin Zhang [1] and Wenju Peng [1,3,*]

[1] School of Chemistry and Materials Science, Hubei Engineering University, Xiaogan 432000, China; 15394082645@163.com (W.Y.); zhengyuanzhu@foxmail.com (Z.Z.); 13415968580@163.com (L.Z.)
[2] School of Materials Science and Engineering, Hubei University, Wuhan 430062, China
[3] School of Civil Engineering, Hubei Engineering University, Xiaogan 432000, China
* Correspondence: yaoyaozhang@hbeu.edu.cn (Y.Z.); pwj1112@foxmail.com (W.P.)

CONTENT：

1. Characterization of the template $PN_{64}(IL)_8$

2. XPS analysis of ZnO-Ce-x‰

3. Raman analysis of ZnO-Ce-x‰

4. Influence of catalyst amount and initial concentration of MO

1. Characterization of the template $PN_{64}(IL)_8$

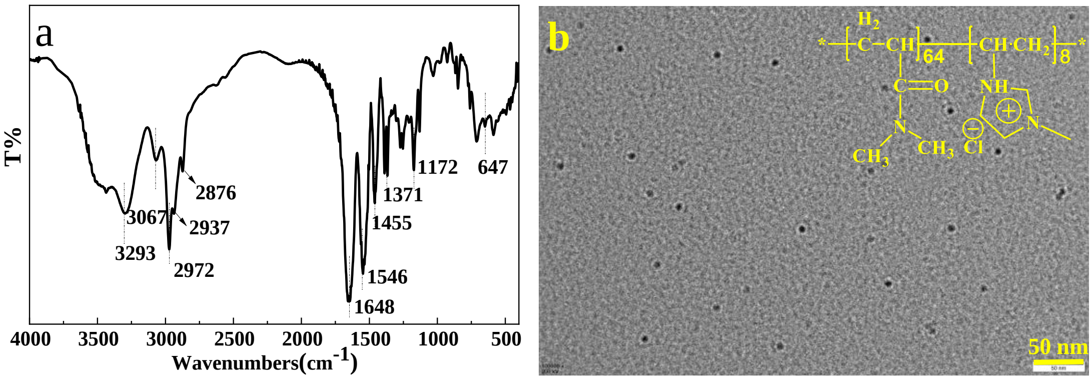

Figure S1. FT-IR spectra of $PN_{64}(IL)_8$ (a), TEM of $PN_{64}(IL)_8$ (b).

As Figure S1 showed, the FT-IR spectra of thermosensitive material template $PN_{64}(IL)_8$ exhibited the characteristic peaks of PNIPAAm at 3293, 3067, 2972, 2937, 2876, 1648, 1546 cm^{-1} (Figure S1a). Among them, the characteristic peaks at 3293 and 3067 cm^{-1} belong to the -NH- group in -CONH-, the peaks at 2972 and 2937 cm^{-1} belong to the -CH- group. $PN_{64}(IL)_8$ showed the characteristic peaks of $-CH(CH_3)_2$ in 2876 cm^{-1}, while 1648 cm^{-1} and 1546 cm^{-1} exhibit the stretching vibration peak of -CH- in $-CH(CH_3)_2$ [1]. The peaks at 1455 cm^{-1} and 1371 cm^{-1} belong to the flexural vibration of $-CH_3-$ group. 1172 cm^{-1} belong to the -C=O- group of NIPAAm. Due to the introduction of ionic liquid unit into the material, 647 cm^{-1} appears ionic liquid characteristic peaks, proved the surface polymer structure contains ionic liquid unit. TEM was used to characterize the morphology of $PN_{64}(IL)_8$. As shown in Figure S1b, the material showed good solubility and uniform dispersion in water with a size of about 5 nm particles.

2. XPS analysis of ZnO-Ce-x‰

In order to investigate the chemical composition and valence states of the elements, the ZnO-Ce-x‰ photocatalytic material was tested by XPS. As shown in Figure S2, O, Zn, and Ce in ZnO-Ce-x‰ materials belong to ZnO and CeO_2, respectively. The binding energy of the C 1s peak was used to calibrate the baseline. Figure S2 shows that the binding energy of Zn in ZnO-Ce-x‰ material is around 1022.1 eV and 1045.0 eV, and the high-resolution XPS spectra of Zn 2p were perfectly fitted, so Zn in the sample exists in the form of Zn^{2+} [2]. The Ce 3d photoelectron peak could be divided into two pairs of spin-orbit

multiplets, with the peak values at 884.7 eV and 903.8 eV, confirming the existence of Ce^{4+} [3]. We provide the XPS of ZnO-Ce-2‰, ZnO-Ce-6‰, ZnO-Ce-10‰, ZnO-Ce-14‰, a quantitative table for the chemical composition percentages (Table 2), and the ratio of the actual Ce loading to Zn is closed to the added content.

Table S1. Atomic percentages of ZnO-Ce-x‰.

	ZnO-Ce-2‰	ZnO-Ce-6‰	ZnO-Ce-10‰	ZnO-Ce-14‰
C1s	43.57	44.55	43.82	44.66
Ce3d	0.03	0.07	0.11	0.17
O1s	44.03	43.25	43.68	43.46
Zn2p	12.37	12.13	12.39	11.71

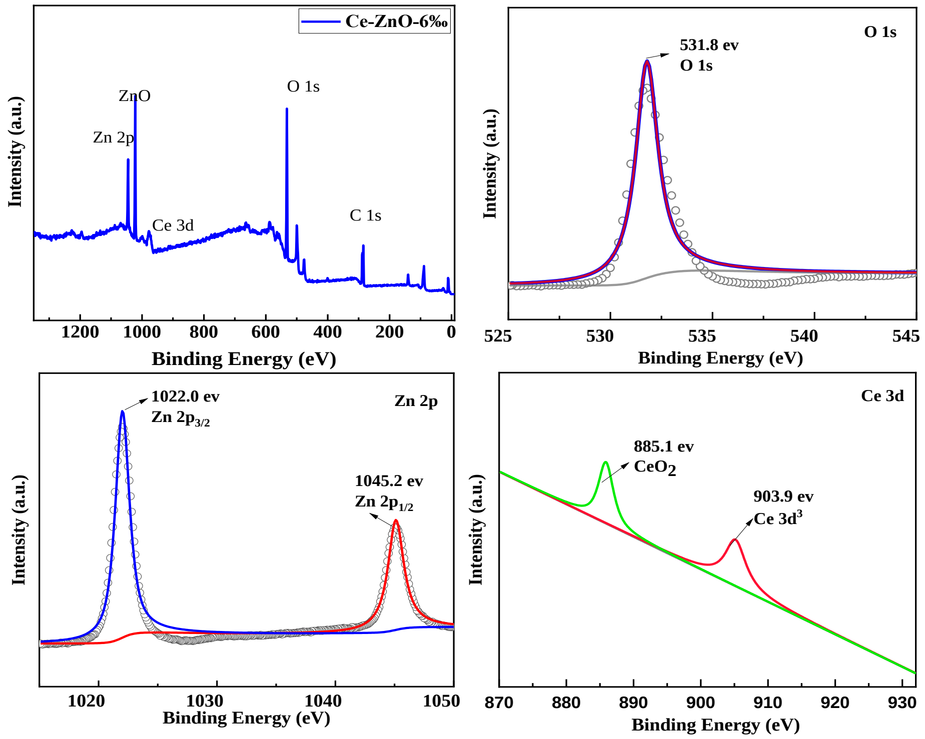

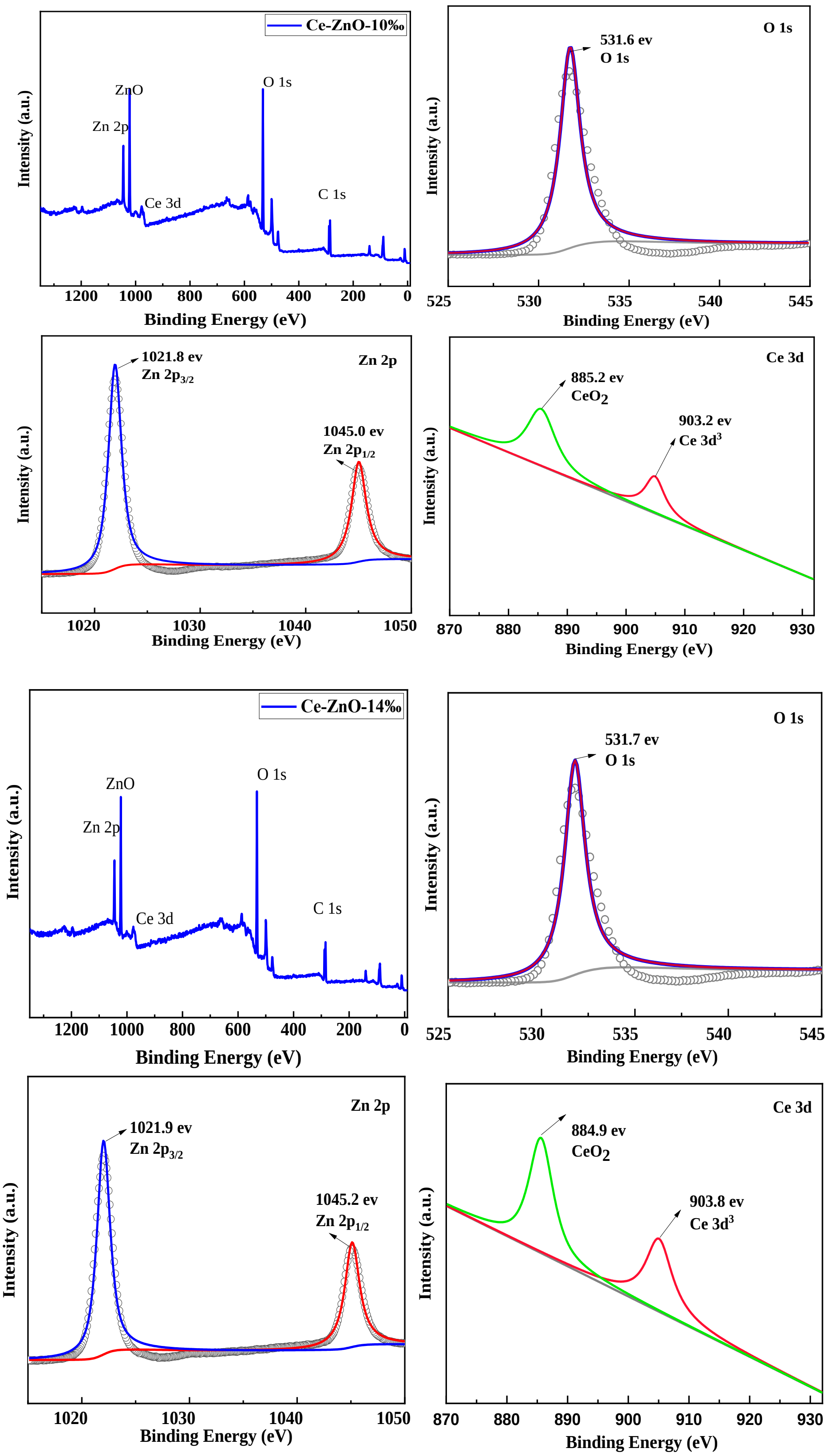

Figure S2. XPS of ZnO-Ce-6‰, ZnO-Ce-10‰, ZnO-Ce-14‰ with the added of template $PN_{64}(IL)_8$.

3. Raman analysis of ZnO-Ce-x‰

Figure S3 shows representative Stokes-side Raman spectra of the ZnO and Ce doped ZnO-Ce-x‰. ZnO has a wurtzite structure, which belongs to the space group C_{6v}^4 with two formula units per primitive cell where all atoms occupy C_{3v}. Zone center optical phonons predicted by group theory are A1+2E2+E1. Here, A1 and E1 modes are polar and split into the transverse optical (TO) and longitudinal optical (LO) phonons. In addition, E2 mode consists of two modes: $E2_{(high)}$ is associated with the vibration of oxygen atoms and $E2_{(low)}$ is associated with the Zn sublattice. The spectra show several peaks characteristic of vibrational modes in ZnO, and all of the characteristic Raman peaks are listed (Table S2). As Figure S3, the E_1(LO) band at 582.8 cm^{-1} of ZnO-Ce-2‰ is manifestation of the resonant enhancement of the LO mode due to the presence of Ce doping defects. The systematic increase of the $E_1(\mathrm{LO})/E_2^{\mathrm{high}}$ is in accord with an increasing defect concentration with adding Ce to form ZnO-Ce-2‰ ($E_1(\mathrm{LO})/E_2^{\mathrm{high}}$=0.0888) than ZnO ($E_1(\mathrm{LO})/E_2^{\mathrm{high}}$=0.0738) by calculation.

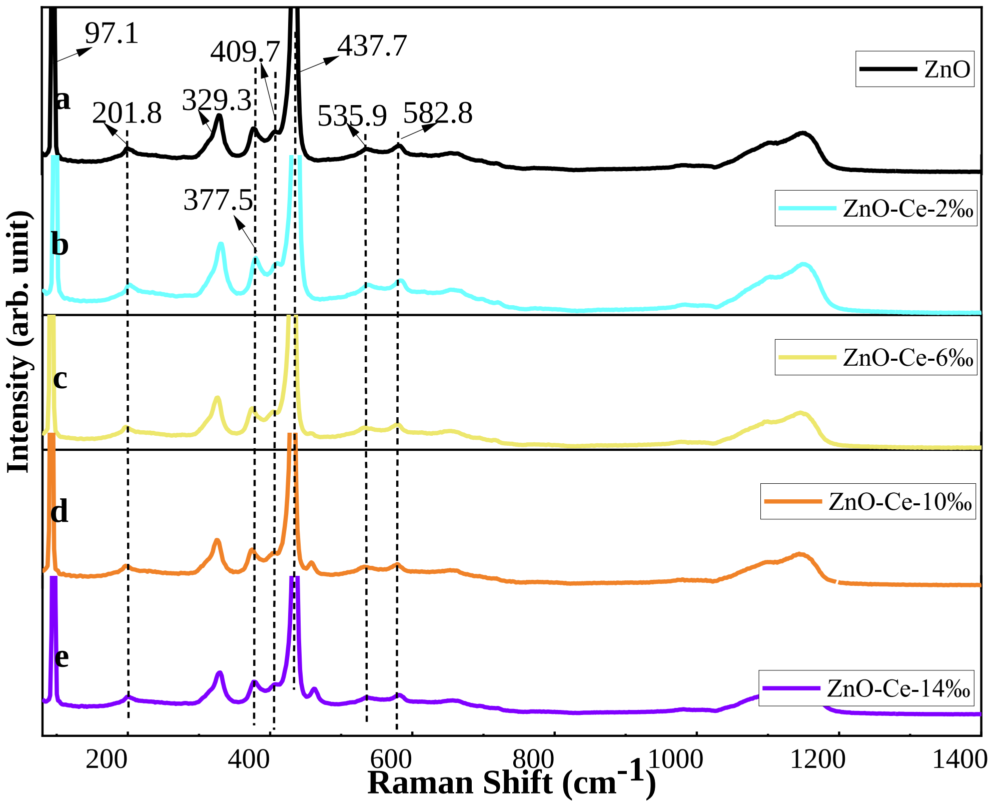

Figure S3. Raman spectra of ZnO (a), ZnO-Ce-2‰ (b), ZnO-Ce-6‰ (c), ZnO-Ce-10‰ (d), ZnO-Ce-14‰ (e).

Table S2. Raman modes of ZnO nanospindles in samples ZnO, ZnO-Ce-2‰, ZnO-Ce-6‰, ZnO-Ce-10‰, ZnO-Ce-14‰.

Peak identity	Present study	Reported	Reported
$2E_2^{low}$	201.8	203	Ref. [4]
E_2^{high}-E_2^{low}	329.3	327.9	Ref. [5]
A_1(TO)	377.5	378	Ref. [4]
E_1(TO)	409.7	410	Ref. [4]
E_2^{high}	437.7	438	Ref. [6]
$2B_1^{low}$	535.9	536	Ref. [5]
E_1(LO)	582.2	590	Ref. [5]

4. Influence of catalyst amount and initial concentration of MO

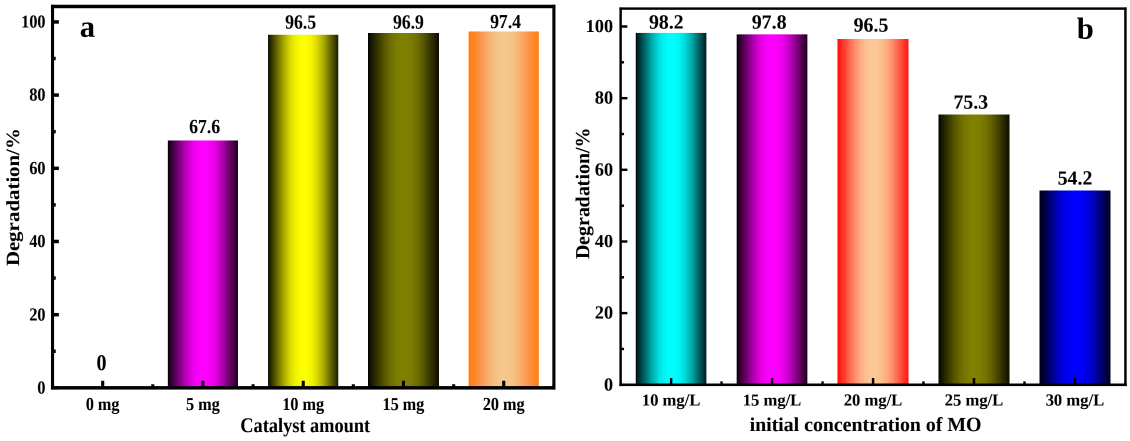

Figure S4. Influence of catalyst amount (a) and initial concentration of MO (b) in the presence of ZnO-Ce-2‰.

Since the optimum catalytic material was ZnO-Ce-2‰, the dosage of the catalytic material ZnO-Ce-2‰ was investigated. The results showed that MO was not degraded in the absence of catalytic material, and a degradation rate of 67.6% was obtained by adding 5 mg ZnO-Ce-2‰. When increased ZnO-Ce-2‰ to 10 mg, it can show higher catalytic performance. Further added the catalytic material, the degradation rate was maintained at about 96.5%, so 10 mg catalyst was the optimum amount of catalyst (Figure S4a). The initial concentrations of MO were then investigated and MO solutions with concentrations of 10 mg/L, 15 mg/L, 20 mg/L, 25 mg/L, and 30 mg/L were prepared. 10 mg of the catalytic material ZnO-Ce-2‰ powder was added to MO solutions of different concentrations for photodegradation experiments. The results showed that at lower concentrations of 10 mg/L, 15 mg/L, and 20 mg/L, the catalytic materials could achieve 98.2%, 97.8%, 96.5% degradation rate, respectively. Even at a higher concentration of 25 mg/L, the degradation

rate up to 75.3%. Further increased the concentration to 30 mg/L, the degradation rate could reach an average of 54.2% (Figure S4b). This work was higher than the general MO concentration reported in the current literature, and the catalytic degradation effect was much better [7–10].

References

1. Zhang, Y.; Zhou, L.; Han, B.; Li, B.; Wang, L.; Wang, J.; Wang, X.; Zhu, L. Controllable preparation of chiral oxazoline-Cu(II) catalyst as nanoreactor for highly asymmetric Henry reaction in water. *Catal. Lett.* **2022**, *152*, 106–115. https://doi.org/10.1007/s10562-021-03633-5.
2. Choudhary, S.; Sharma, M.; Krishnan, V.; Mohapatra, S. Facile synthesis of Ce doped ZnO nanowires for efficient photocatalytic removal of organic pollutants from water. *Mater. Today Commun.* **2023**, *34*, 105361. https://doi.org/10.1016/j.mtcomm.2023.105361.
3. Lamba, R.; Umar, A.; Mehta, S.K.; Kansal, S.K. CeO_2-ZnO hexagonal nanodisks: Efficient material for the degradation of direct blue 15 dye and its simulated dye bath effluent under solar light. *J. Alloys Compd.* **2015**, *620*, 67–73. https://doi.org/10.1016/j.jallcom.2014.09.101.
4. Cuscó, R.; Alarcón-Lladó, E.; Ibáñez, J.; Artús, L. Temperature dependence of Raman scattering in ZnO. *Phys. Rev.* **2007**, *75*, 165202..
5. Giri, P.K.; Bhattacharyya, S.; Singh, DK.; Kesavamoorthy, R.; Panigrahi, B.K.; Nair, G.M. Correlation between microstructure and optical properties of ZnO nanoparticles synthesized by ball milling. *J. Appl. Phys.* **2007**, *102*, 093515.
6. Aggelopoulos, C.A.; Dimitropoulos, M.; Govatsi, A.; Sygellou, L.; Tsakiroglou, C.D.; Yannopoulos, S.N. Influence of the surface-to-bulk defects ratio of ZnO and TiO_2 on their UV-mediated photocatalytic activity. *Appl. Catal. B Environ.* **2017**, *205*, 292–301.
7. Gherbi, B.; Laouini, S.E.; Meneceur, S.; Bouafia, A.; Hemmami, H.; Tedjani, M.L.; Thiripuranathar, G.; Barhoum, A.; Menaa, F. Effect of pH value on the bandgap energy and particles size for biosynthesis of ZnO nanoparticles: Efficiency for photocatalytic adsorption of methyl orange. *Sustainability* **2022**, *14*, 11300. https://doi.org/10.3390/su141811300.
8. Hannachi, E.; Slimani, Y.; Nawaz, M.; Sivakumar, R.; Trabelsi, Z.; Vignesh, R.; Akhtar, S.; Almessiere, M.A.; Baykal, A.; Yasin, G. Preparation of cerium and yttrium doped ZnO nanoparticles and tracking their structural, optical, and photocatalytic performances. *J. Rare Earths* **2023**, *41*, 682–688. https://doi.org/10.1016/j.jre.2022.03.020.
9. Bao, L.-L.; Li, Y.; Xi, Z.; Wang, X.-Y.; Afzal, M.; Alarifi, A.; Srivastava, D.; Prakash, O.; Kumar, A.; Jin, J.-C. A new 2D Zn(II)-based coordination polymer as photocatalyst for photodegradation of methyl orange in water: Effect of photocatalyst dosage and dye concentration. *J. Mol. Struct.* **2023**, *1292*, 136103. https://doi.org/10.1016/j.molstruc.2023.136103.
10. Gilani, S.A.B.; Naseeb, F.; Kiran, A.; Ihsan, M.U.; Iqbal, J.; Javed, H.M.A.; Bhatti, H.N.; Karami, A.M.; Hussain, S.; ShabirMahr, M. pH dependent synthesis of ceria nanoparticles for efficient sunlight-driven photocatalysis of methyl orange containing wastewater. *Opt. Mater.* **2024**, *148*, 114871. https://doi.org/10.1016/j.optmat.2024.114871.
